# Neuroprotective effects of dantrolene in neurodegenerative disease: Role of inhibition of pathological inflammation

**DOI:** 10.1016/j.jatmed.2024.04.002

**Published:** 2024-04-30

**Authors:** Wenjia Zhang, Xu Zhao, Piplu Bhuiyan, Henry Liu, Huafeng Wei

**Affiliations:** a Department of Anesthesiology and Critical Care, Perelman School of Medicine, University of Pennsylvania, Philadelphia, PA 19104, USA; b Department of Anesthesiology, Shandong Provincial Hospital, Shandong First Medical University, Jinan, Shandong 250021, China

**Keywords:** Alzheimer’s disease, Cognitive dysfunction, Progressive motor dysfunction, Apoptosis, Calcium, Pyroptosis

## Abstract

Neurodegenerative diseases (NDs) refer to a group of diseases in which slow, continuous cell death is the main pathogenic event in the nervous system. Most NDs are characterized by cognitive dysfunction or progressive motor dysfunction. Treatments of NDs mainly target alleviating symptoms, and most NDs do not have disease-modifying drugs. The pathogenesis of NDs involves inflammation and apoptosis mediated by mitochondrial dysfunction. Dantrolene, approved by the US Food and Drug Administration, acts as a RyRs antagonist for the treatment of malignant hyperthermia, spasticity, neuroleptic syndrome, ecstasy intoxication and exertional heat stroke with tolerable side effects. Recently, dantrolene has also shown therapeutic effects in some NDs. Its neuroprotective mechanisms include the reduction of excitotoxicity, apoptosis and neuroinflammation. In summary, dantrolene can be considered as a potential therapeutic candidate for NDs.

## Introduction

Neurodegenerative diseases (NDs) refer to a group of diseases characterized by progressive and chronic cell death within the nervous system, manifesting clinically as symptoms reflective of the pathophysiological changes caused by neuronal loss.^1^ Many diseases can be classified into NDs. The prototypical examples include Parkinson’s disease (PD), Alzheimer’s disease (AD), Huntington’s disease (HD), amyotrophic lateral sclerosis (ALS), and multiple sclerosis (MS). Aging is the biggest risk factor for most NDs.^2^ Common NDs and their estimated prevalence, as reported in primary literature, are shown in Table 1.

Most NDs are characterized by cognitive dysfunction or progressive motor dysfunction, thus placing an increasing burden on caregivers and economy. More importantly, PD stands as the only treatable ND currently with the individualized treatment objectives, emphasizing the need of personalized management. Levodopa serves as the primary first-line treatment. Achieving optimal treatment begins with diagnosis and requires a multidisciplinary approach that includes a number of nonpharmacological interventions.^7^ All other NDs are incurable. To date, two types of drugs have received approval for AD, including cholinesterase inhibitors and the N - methyl - D - aspartate (NMDA) receptor antagonist. However, these medications only alleviate the symptoms of AD and do not provide a cure or preventive measures against the disease itself.^8^ Potential interventions for HD include therapies targeting huntingtin DNA and RNA, clearance of huntingtin protein, DNA repair pathways, and other treatment strategies targeting inflammation and cell death.^9^ For ALS, survival can be improved through supportive and symptomatic care delivered by a multidisciplinary team of experts. This approach may involve expert consensus guidelines and an evidence-based approach to treatment.^10^ The greatest challenge in MS lies in developing therapies that combine neuroprotection and remyelination to treat, and ultimately disable, the progression of the disease.^11^ Currently, many disease-modifying therapies for patients in early aggressive courses have shown variable efficacy in preventing recurrence, lesion accumulation on magnetic resonance imaging (MRI), and disability progression.^12^

Dantrolene, a Food and Drug Administration (FDA)-approved drug for the treatment of malignant hyperthermia, has recently been evaluated for prospective use as a neuroprotective agent for the treatment of neurodegenerative syndromes including AD.^13^ Researchers have demonstrated that dantrolene improves memory loss and neuropathology in different animal models of AD.^14,15^ Through in-vitro experiments, researchers have also demonstrated that dantrolene ameliorated the impairments in neurogenesis and synaptogenesis.^16^ Recent studies also show dantrolene’s therapeutic effects in different NDs including AD^14,15^ and HD.^80^ The potential mechanisms by which dantrolene protects neurons are decreasing excitotoxicity, apoptosis, and neuroinflammation.

## Pathogenesis of NDs and potential therapeutic pathways

Although NDs are heterogeneous and contain a series of different diseases, the study of genetic defects and disease-related proteins indicates that different NDs share similar features, mechanisms, and even genetic or protein abnormalities.^17^

There are two main mechanisms that may explain this challenging process. One is an inflammation-mediated immune response, and the other is apoptosis, a form of programmed cell death. The two mechanisms are implicated in the pathogenesis of NDs, including AD, PD, HD, MS, and ALS.^18,19^

To date, mitochondrial dysfunction has been thought to contribute to inflammation and apoptosis in NDs through the accumulation of mitochondrial DNA (mtDNA) mutations and the generation of reactive oxygen species (ROS).^17,20^ Specific interactions of disease-related proteins with mitochondria have recently been uncovered: APP, β-amyloid (Aβ), presenilin, α-synuclein, parkin, DJ-1, PINK1 (phosphatase and tension homolog-induced kinase 1), LRRK2 (leucine-rich-repeat kinase 2), HTRA2, SOD1 and huntingtin have all been demonstrated to localize within the mitochondria.^20,21^

The pathology of inflammation and mitochondrial dysfunction-mediated apoptosis in AD is characterized by the accumulation of amyloid β-containing neurotic plaques, neurofibrillary tangles, and dystrophic neurites containing hyperphosphorylated tau protein. Microglia, the resident macrophages in the brain, become activated by pathological events such as neuronal death or protein aggregates. Once activated, they can bind to Aβ oligomers and Aβ fibrils via cell surface receptors. SCARA1, CD36, CD14, α6β1 integrin, CD47, and Toll-like receptors (TLR2, TLR4, TLR6, and TLR9) are receptors. The process of microglia activation is thought to be part of the inflammatory reaction in AD.^22^ Inefficient clearance of Aβ has been identified as a major pathogenic pathway in sporadic cases of AD.^22^ By decreasing the expression of Aβ phagocytosis receptors, microglia show insufficient phagocytic capacity which is thought to be responsible for increased cytokine concentration. Reduced mitophagy process enhances neuroinflammation due to reduced phagocytosis and over stimulation of microglia, leading to increased accumulation of Aβ plaques and hyperphosphorylated tau protein.^22,23^ In addition, the researchers showed that in TgAPPsw and PSAPP transgenic mice, the increases of Aβ concentration were highly correlated with increased concentration of proinflammatory cytokines, including TNFα, interleukin 6 (IL-6), interleukin 1α (IL-1α), and granulocyte-macrophage colony stimulating factor (GM-CSF).^22^

Oxidative damage in the brain of AD transgenic APP mice proceeds the occurrence of Aβ deposition and significant plaque pathological changes. The signaling pathways of APP or tau processing may be activated by oxidative stress. In vitro, studies have shown that exposure to Aβ increases oxidative stress and decreases energy availability, thereby increasing the susceptibility of cell death by inducing apoptosis. However, evidence regarding the role of apoptosis in neuronal cell death in AD remains limited.^24,25^

Inflammation and mitochondrial dysfunction-mediated apoptosis are important pathologies of PD. The pathological feature of PD is the presence of Lewy bodies, resulting from α-synuclein aggregation and dopaminergic neurodegeneration in the substantia nigra pars compacta. Dopaminergic neurons are selectively vulnerable to mitochondrial dysfunction. Subsequently, the mitochondrial PT pore opens, resulting in the release of cytochrome c into the cytoplasm, triggering the mitochondria-mediated apoptotic pathway, which is considered to be the primary mechanism of dopaminergic neuronal death in PD. MPTP (1-methyl 4-phenyl-1,2,3,6-tetrahydropyridine), whose metabolite MPP+ inhibits complex I of the mitochondrial electron-transport chain, causing parkinsonism in designer-drug abusers.^26,27^

Another important pathogenesis is pro-inflammatory immune-mediated mechanisms. Results from animal models confirmed the activation of microglia and the upregulation of pro-inflammatory mediators such as cytokines found in postmortem PD brains.^18^ Antigens such as infectious pathogens, prions, pathologically modified proteins, aggregates, and apoptotic cells can activate microglia. Activated microglia subsequently secrete a wide range of inflammatory mediators, such as TNFα, IL-6, nitric oxide synthase 2 (NOS2), COX2, and ROS, leading to cell proliferation, followed by slow degeneration, and finally the death of dopaminergic neurons.^28^ Furthermore, clinical studies in PD patients examined the CSF and peripheral blood, showing elevated serum IL-1, IL-6, and TGF-β in CSF.^29^ Recently, mitochondrial dysfunction has been proven to induce neuroinflammation.^18^

Inflammation and mitochondrial dysfunction also mediate apoptosis in HD. HD is caused by mutations in an abnormally expanded CAG trinucleotide repeat in a gene encoding the polyglutamine repeats in the huntingtin protein. Inflammation in HD can be triggered by glial cells responses to autonomous neuronal degeneration or immune cells activation due to mHtt (mutant HTT protein) expression, or both at the same time.^30^ The main inflammatory processes are carried out by activated microglia and astrocytes. Reactive astrocytes are observed in presymptomatic HD and correlate with disease severity.^31^

Studies have shown that the activity of complexes II and III of the electron-transport chain is reduced in the brains of HD patients. Additionally, mitochondrial respiration and ATP production were also significantly impaired in striatal cells in mutant HTT-knock-in mouse embryos. Moreover, cytochrome c discharge, BAX overexpression, and active caspase-3, −8, and −9 have been illustrated in the brains of HD patients and in experimental models of HD.^32^ These evidence suggest that mitochondrial dysfunction-mediated apoptosis plays an important role in the pathogenesis of HD.

Inflammation and mitochondrial dysfunction mediate apoptosis in ALS. ALS is recognized by the progressive and specific loss of motor neurons in the cortex and the ventral horn of the spinal cord. Studies in ALS patients and animal models have shown that activated microglia and astroglia, proinflammatory peripheral lymphocytes, and macrophages are associated with ALS.^33^ The anti-inflammatory response is initially protective, but subsequently, inflammatory cytotoxic cytokines (IL-1β, IL-6, IL-18, TNF-α etc.) and reactive oxygen species (ROS) can lead to cell death and further tissue damage.^34^ In recent years, an increasing number of studies have revealed that both CNS and peripheral immune cells release extracellular vesicles, which can regulate the behavior of neighboring receptor cells and play an essential role in the pathogenesis of ALS.^33^

The spinal cord, nerve, and muscle biopsy samples showed abnormal mitochondria structures and numbers. The study focused on the expression of mutant SOD1 in animal and cellular models of ALS. In fact, SOD1 plays a vital role in the clearance of ROS, and the abnormal activity of mutant human SOD1 (mSOD1) leads to oxidative damage.^35^ Transgenic mice overexpressing the G93A *Sod1* mutation exhibit impaired mitochondrial energy metabolism in the brain and spinal cord during disease onset. The illustration of DNA fragmentation has suggested the role of apoptosis in ALS pathogenesis, Caspase 9 activation, cytochrome c discharge, BAX overexpression, and reduced Bcl-2 expression in postmortem tissue and transgenic mouse models of ALS.^36–38^

Inflammation and mitochondrial dysfunction mediated apoptosis in MS. The pathological hallmark of all MS phenotypes is focal plaques (also called lesions), which are areas of demyelination typically located around post-capillary venules and characterized by breakdown of the blood-brain barrier (BBB). The mechanisms underlying BBB breakdown are not fully understood but seem to involve direct action of pro-inflammatory cytokines and chemokines (such as TNF-α, IL-1β, and IL-6) produced by resident and endothelial cells, as well as indirect cytokine-dependent and chemokine-dependent leukocyte-mediated damage.^39^ BBB dysregulation increases the trans-endothelial migration of activated leukocytes, including macrophages, T cells, and B cells, into the CNS. This process leads to further inflammation and demyelination, resulting in oligodendrocyte loss, reactive gliosis, and neuro-axonal degeneration.^39^

In addition to inflammatory mechanism, researchers are currently focusing on the role of mitochondrial damage in demyelination and neurodegeneration. Biochemical studies identified impaired NADH dehydrogenase activity and increased complex IV activity in mitochondria in MS lesions.^40^ Furthermore, in oligodendrocytes, mitochondrial damage results in the release of apoptosis-inducing factor, its translocation into the nuclei, and the activation of poly-ADP-ribose polymerase (PARP), a mechanism demonstrated in oligodendrocyte destruction and demyelination induced by cuprizone intoxication in an experiment in vivo.^40^

## Therapeutic pathways

Given the growing evidence supporting the role of inflammation and mitochondrial dysfunction-mediated apoptosis in the pathogenesis of various NDs, treatments involving anti-inflammation and improving mitochondrial function may serve as potential therapeutic approaches.

### Immunomodulatory therapies

Experimental evidence and animal studies showed that non-steroidal anti-inflammatory drugs (NSAIDs), particularly ibuprofen and piroxicam have promising results and appeared to reduce PD risk.^18^ Other potential immunomodulatory therapies, such as anti-TNF therapies, are based on in vitro studies and need more in vivo evidence.^18^ Drugs such as PPARγ agonists, COX inhibitors, vitamin E, vitamin C, curcumin and catechin, which reduce the neuroinflammatory pathways, are thought to prevent the progression of AD.^23^ Preclinical studies in HD mouse models have shown that active immunity is achieved by the introduction of an immunogenic short peptide. This peptide generates host B cell-mediated antigen-specific antibodies against exon1 of mutant HTT protein. Consequently, this approach downregulates aberrant neuroinflammatory and cell death pathways.^31^ Minocycline, which can suppress microglial activation and modulate apoptosis, was shown to reduce motor neurons loss, delay disease onset, and extend the survival of SOD1^G93A^ mice. NP001, which has been shown to be safe and well tolerated, can slow disease progression by modulating monocyte activation and down-regulating NF-kB in macrophages.^41^

### Inhibition of mitochondrial cytochrome c release

Minocycline can also inhibit mitochondrial cytochrome c release, which is an essential step in the progression of the mitochondria-mediated apoptotic pathway. Moreover, it displays broad neuroprotection in experimental models of NDs.^42,43^

### The use of antioxidants

One of the most studied antioxidant compounds is mitoquinone (MitoQ), which exerts direct antioxidant action by scavenging superoxide, peroxyl, and peroxynitrite ROS.^44^ In an AD transgenic mouse model expressing three human mutant genes, APP, PSEN1 and tau, MitoQ treatment showed improved behavioral phenotype. Other anti-oxidants, such as Skulachev (SkQ1), MitoApo, melatonin, have also been reported to show neuroprotective effects in different research models.^45–48^

### Overexpression of the anti-apoptotic protein Bcl-2

CGP 3466 is a drug that ultimately results in the upregulation of anti-apoptotic Bcl-2. CGP 3466 has been reported to rescue dopaminergic neurons from death and to subsequently inhibit the development of motor symptoms in rodent models of PD by preventing mitochondria-mediated apoptosis.^49^ Additionally, Bcl-2 overexpression and the deletion of pro-apoptotic BAX and BAK have been shown to exert neuroprotection in ALS mice by blocking the apoptosis of lumbar spinal motor neurons and delaying the onset of symptoms.^50^

## Dantrolene pharmacology

Dantrolene is the only effective treatment for malignant hyperthermia (MH), a fatal condition during general anesthesia. Before the advent of dantrolene, the mortality rate of MH was reported as high as 80 %.^51^ Dantrolene was approved by the Food and Drug Administration (FDA) in 1979, and can effectively reverse the symptoms and reduce the mortality to less than 5 %.^52^

## Mechanism of action

The ryanodine receptors (RyRs) are major intracellular Ca ^2+^ release channels located in the plasma membrane of the endoplasmic/sarcoplasmic reticulum. Three mammalian isoforms of RyRs have been isolated. Type 1 RyRs (RyR-1) is preferentially expressed in skeletal muscle and in cerebellar Purkinje neurons. Type 2 RyRs (RyR-2) is predominantly expressed in cardiac muscle and is the most abundant isoform in the brain. Type 3 RyRs (RyR-3) was first identified in the brain and is mainly found in cortical and hippocampal regions involved in learning and memory.^53^ Both RyR-1 and RyR-3 are the targets of dantrolene in vivo, while previously RYR2 in cardiac SR vesicles was thought insensitive to clinical concentrations of dantrolene.^54^ A recent in vitro study shows that the inhibition of dantrolene to RyR2 complex is very sensitive but depends on the presence of both FKBP12.6(a regulatory protein) and calmodulin (CaM).^55^

The hypermetabolic symptoms of MH are due to the abnormal release of the Ca^2+^ from the sarcoplasmic reticulum (SR) via RyR-1 in skeletal muscles.^56^ Symptoms of chronic ischemia heart disease including ventricular tachycardia, left-ventricular remodeling, and contractile dysfunction are highly associated with the RyR-2 hyper-activity.^57^ Dantrolene is a ryanodine receptor antagonist that acts directly on the RyR-1 and RyR-3 to reduce channel activation by CaM. Thereby it decreases the high Ca^2+^ concentration that leads to sustained pathological muscle contraction.^51,53,58^ Dantrolene also stabilizes RyR-2 tetrameric structure and improves survival after myocardial infarction.^59^

## Chemistry and pharmacokinetics

Dantrolene is highly lipophilic and therefore poorly soluble in water. Dantrolene can now be administered intravenously. One bottle of 20 mg dantrolene sodium contains 3 g mannitol to improve water solubility. The powder should be dissolved in 60 ml water to achieve a concentration of 0.33 mg/ml, and adjusted the final pH to 9.5.

In 2014, FDA approved Ryanodex, a new IV formulation of dantrolene nanoparticles. Ryanodex (Eagle)^60^ contains 250 mg of dantrolene as a lyophilized powder. Each vial of powder requires only 5 ml of sterile water (SWI) to reconstitute for injection, while other previously approved formulations required 60 ml of SWI to reconstitute a 20 mg dose. Ryanodex contains less mannitol, so patients may need to supplement with mannitol to produce more urine.^60^

Approximately 70 % of dantrolene is absorbed after oral administration, with a peak plasma time of six hours. However, plasma concentration varies significantly from patient to patient, especially in children.^61^ The plasma elimination half-life is estimated to be 12 h. In children, the pharmacokinetic profile is similar, with a half-life of approximately 10 h.^62^ Hepatic microsomes metabolize Dantrolene to 5-hydroxydantrolene. Dantrolene and its metabolites are mainly excreted through urine and bile.^63,64^

## Therapeutic uses

In addition to malignant hyperthermia, dantrolene is also used to treat other diseases in clinical work.

## Muscle spasm

The first approved use of oral dantrolene was for the treatment of spasticity.^65^ Spasticity is a common symptom of many neurological disorders, such as cerebral infarction and MS.^66^ Non-drug treatments for spasticity include passive movements, exercises, posture and standing. Commonly used anti-spasmodic drugs are those that act on the gamma aminobutyric acid (GABA) energic system (baclofen, gabapentin, and benzodiazepines), the α-2 adrenergic system (tizanidine) Unlike other drugs that treat spasm, dantrolene works directly on the muscle and so is less sedative.

## Neuroleptic malignant syndrome

The incidence of neuroleptic malignant syndrome (NMS) has been reported to be rare, and the most obvious clinical features of NMS are muscle rigidity and hyperthermia. As the cause of altered thermo-regulation remains unclear, skeletal muscle contraction may generate heat. Therefore, paralytics will be a key cooling measures.^67^ Dantrolene, along with dopamine agonists such as bromocriptine and amantadine, may provide benefits. However, these findings are solely based on case reports due to the absence of controlled clinical trials.^68^

## Ecstasy intoxication

One of the crucial signs of ecstasy intoxication is hyperthermia, which may be caused by overstimulation of central serotonin. Dantrolene has been proposed as a potentially effective treatment for ecstasy poisoning in emergency rooms. However, recent animal studies suggest that dantrolene sodium formulations are mechanistically unsuitable for the treatment of MDMA- and METH-induced hyperthermia.^69^

## Exertional heat stroke

Exertional heat stroke (EHS) is an acute injury with high morbidity and mortality that is common in military and special operations environments. The most effective way to treat EHS is immediately cooling. Dantrolene is administered to the victim to prevent further muscle contractions and hyperthermia. However, these data come from case reports. Whether dantrolene treatment is beneficial for EHS still requires randomized, controlled clinical studies.^70^

## Adverse effects

Adverse effects may occur following acute or chronic parenteral administration of dantrolene. The most common side effect was muscle weakness (22 %), followed by phlebitis (10 %), respiratory failure (3 %), and gastrointestinal discomfort (3 %). Other adverse symptoms of dantrolene treatment include drowsiness, dizziness, and confusion.^61^ Long-term oral therapy at high dose has been associated with liver dysfunction, but dantrolene is not the only potentially hepatotoxic substance administered to patients reported to have this complication.^65^

## Dantrolene in neurodegenerative diseases

### Dantrolene’s anti-inflammation effect

In recent years, research on application of dantrolene in different diseases has been studied, including in vitro cell models and in vivo animal models. Recent evidence suggests that disruption of intracellular Ca^2+^ homeostasis resulting from overreaction of RyRs on the ER and/or SR, plays a crucial role in sepsis and COVID-19-induced inflammation.^71,72^ Dantrolene, as a RyRs antagonist, has been expected to improve SARS-CoV-2-mediated inflammation and inhibit SIRS during sepsis by suppressing the concentration of IL-6, IL-8, IL-1β, TNF-α, and IFN-γ3 in plasma and tissues.^71,72^

Wenk and his colleagues infused LPS, a commonly used inflammation inducer, into the brain of 3-months-old F-344 AD rat for 28 days. At the beginning of the inflammation induction, rats were treated subcutaneously with dantrolene (5 mg/kg/day) or nimodipine (5 mg/kg/day). They found that treatment with nimodipine or dantrolene restored the number of TH-immunoreactive cells in the locus coeruleus (LC) and significantly reduced microglia activation in the hippocampus^73^ and substantia nigra pars compacta.^74^ Furthermore, dantrolene treatment decreased inflammatory gene expression of TLR4, iNOS, and TGFβ in the hippocampus, resulting in neurotoxicity.^73^

In another study of autoimmune disease, Fomina’s team used a mouse model of experimental autoimmune encephalomyelitis (EAE), a T cell-mediated autoimmune neuroinflammatory disease. They found that daily intraperitoneal injection of dantrolene at 5 or 10 mg/kg beginning at the time of EAE induction significantly reduced dampened inflammation in the spinal cord.^75^ These findings indicate that dantrolene has anti-inflammation effects in CNS.

### Dantrolene’s therapeutic effects in AD and HD

In 2012, Professor Wei’s lab first reported the use of dantrolene for early and long-term treatment in a murine AD model. Their team published a series of articles on long-term treatment with dantrolene in both 3xTg and 5XFAD mouse models.^14,15^ Their research also revealed that dantrolene reduced amyloid accumulation in neurons by up to 76 % compared to the vehicle control and tended to reduce hippocampal phosphorylated tau protein.^76^ Their studies showed that dantrolene can ameliorate cognitive dysfunction in vivo and has tolerable side effects with long-term use (treatment up to 10 months).^14^ In in vitro experiments on pluripotent stem cells, they found that dantrolene could ameliorates the impairment of neurogenesis and synaptogenesis.^16^

In recent years, impaired neural-lysosomal and autophagy-mediated degradation of cellular debris has been suggested to contribute to neuritic dystrophy and synaptic loss. Mustaly-Kalimi’s group demonstrated that dantrolene can restore autophagic clearance of intracellular protein aggregates by increasing vATPase levels, lysosomal acidification, and proteolytic activity in human iPSC-derived neurons from AD patients. Their work showed that abnormal upstream Ca^2+^ dysregulation contributes to the pathological accumulation of intracellular protein aggregates before the development of overt histopathological or cognitive deficits in AD.^77^ Studies also found that dantrolene preserved synaptic plasticity. Zhang et al. used PS1-M146V knock-in (KI) FAD mice with impaired long-term LTP maintenance (L-LTP). They found that dantrolene could reverse insufficient mushroom spines maintenance in KI neurons.^78^

Besides these exciting findings, there are also controversial results. A study genetically inhibiting RyRs expression in APPPS1 mice showed that blocking RyR-3 was beneficial in older AD neurons while causing more synaptic dysfunction in young AD neurons.^56^

Altogether, these studies of dantrolene in AD are inspiring, but the mechanisms by which dantrolene as a neuroprotective agent still require further investigation.

HD is a progressive neurodegenerative disease caused by polyglutamine amplification in the Huntington protein, resulting in selective degeneration of spine neurons in the striatum. Bezprozvanny’s team fed yeast artificial chromosome transgenic mice (YAC128), a model of HD, dantrolene (5 mg/kg) twice a week from 2 months to 11.5 months of age. Their results showed that the treatment group performed significantly better on beam-walking and gait-walking tests. Furthermore, they performed neuropathological analysis on YAC128 mice and found that the loss of NeuN-positive striatal neurons was significantly reduced after long-term feeding of dantrolene.^79^

Michael S and his colleagues combined whole-cell patch clamp and Two-Photon Ca^2+^ Imaging to record action potential-evoked Ca^2+^ transients in cortical pyramidal neurons (CPNs) in the R6/2 mouse model of juvenile HD at different stages of disease progression. They demonstrated that in the presence of dantrolene, the amplitude and area of Ca^2+^ transients were significantly reduced compared with WT CPNs. The results suggest that dantrolene is a potential therapeutic agent for HD.

### Potential mechanism of dantrolene’s neuroprotective effects

**Fig. 1.**
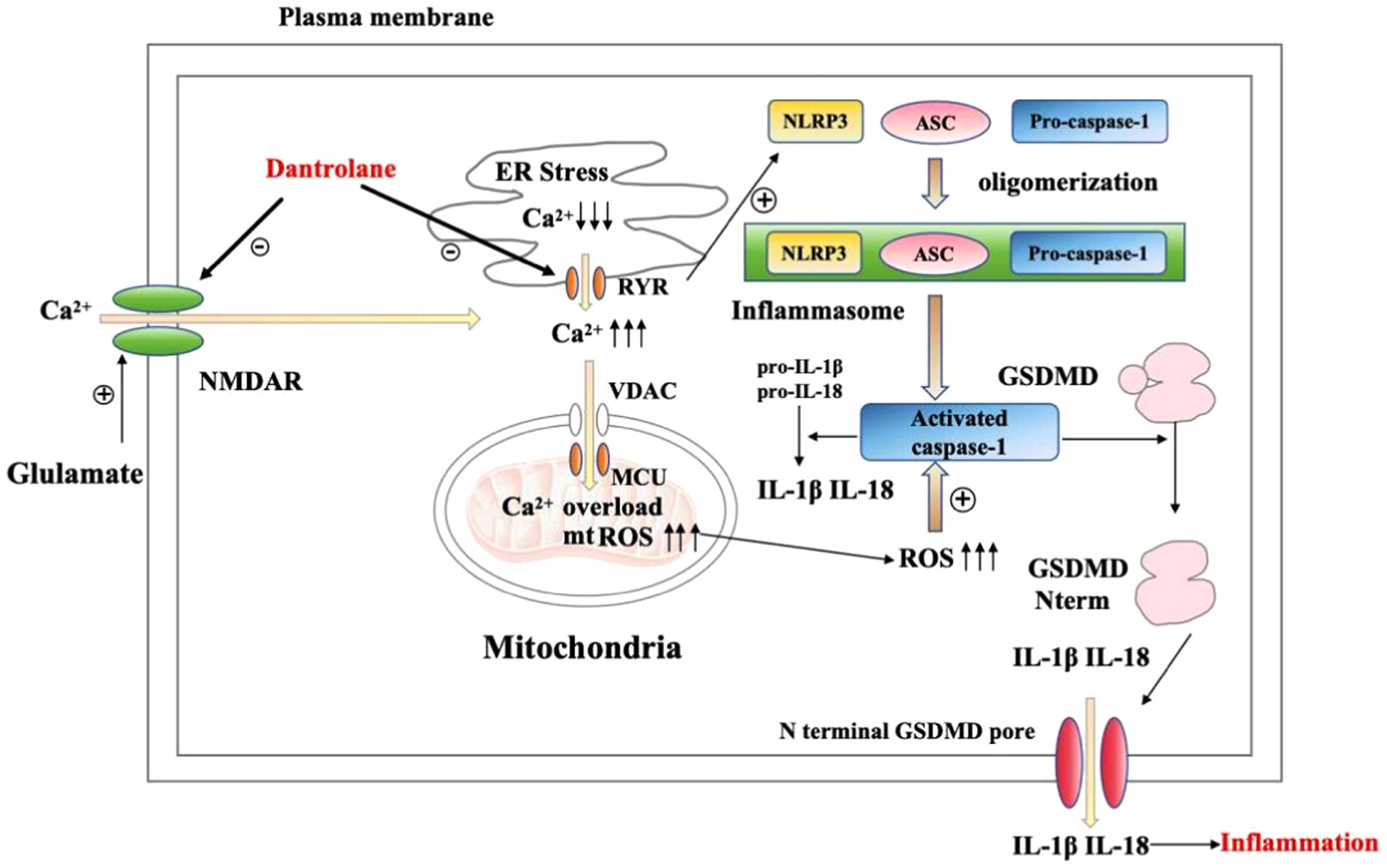
Pyroptosis. Glutamate excitotoxicity result in pathological and excessive activation of NMDAR and/or AMPAR, leading to pathological Ca2+ influx into cytosol space, increasing cytosolic and mitochondria Ca2+ concentration, mitochondrial reactive oxygen species (ROS) and then cytosol ROS. Over activation of RyR results in excessive Ca2+ release from ER and subsequent ER stress. The upstream Ca2+ dysregulation eventually results in activation of NLRP3 inflammasome, activated caspase-1 and cleaved GSDMD to generate N terminal GSDMD and releasing IL-1beta and IL-18 to generate pathological inflammation.

As shown in Fig. 1, RyRs are located on the sarco/endoplasmic reticulum (SR/ER) of nearly all cell types. As we summarized previously, the mechanism of NDs is cell death caused by mitochondrial dysfunction. Increasing studies show that aberrant Ca^2+^ release from intracellular stores (SR/ER) is thought to be an upstream cause of mitochondrial dysfunction. Therefore, aberrant Ca^2+^ release from intracellular stores appears to be a key point.

Calcium is an extremely important signaling ion, transmitting cell signals for various external stimuli. Under physiological conditions, more than 99% of intracellular Ca^2+^ is bound to cytosolic proteins or stored in the ER. Free cytosolic Ca^2+^ is normally maintained at approximately 100 nm, but stimulation can lead to an overall increase to approximately 1 μM.^81^ The mechanisms by which free cytosolic Ca^2+^ rises are Ca^2+^ entry through plasma membrane channels and intracellular pools, in which ER plays an important role. As early as 1961, Engstrom’s group first discovered that mitochondria could rapidly and efficiently take up large amounts of Ca^2+^ when exposed to Ca^2+^ pulses.^82^ In resting neurons, mitochondrial total Ca^2+^ and free Ca^2+^ levels are low, estimated to be approximately 0.1 mM and 100 nM, respectively. However, upon stimulation, mitochondria are able to accumulate enormous amounts of Ca^2+^.^83,84^ Elevated Ca^2+^ in mitochondria has many physiological effects, which are especially important for cellular function. When Ca^2+^ concentration increases, aerobic ATP production will be adjusted, and ROS production will be regulated accordingly. Additionally, elevated Ca^2+^ contributes to synaptic transmission and excitability, organelle dynamics, and activation of the release of death signals.^85,86^ Accordingly, dantrolene (a RyRs inhibitor), has been shown to block the release of ER Ca^2+^ stores and partially protect neurons from oxygen-glucose deprivation toxicity.^87^ Inhibiting the release of ER Ca^2+^ stores may be one of the important mechanisms of dantrolene’s neuroprotective effects.

Chronic inflammation leads to excess Ca^2+^ to be transferred from the ERs to mitochondria, causing mitochondrial calcium overload and further damaging mitochondria.^88^ Moreover, calpains are intracellular Ca^2+^- dependent cysteine proteases that play a physiologic role by cleaving several substrates. Overactivation of calpains can lead to changes in hippocampal structure and function, and is linked to neuronal death. Furthermore, calpain is overactivated by increased Ca^2+^ concentrations.^89^ Hence, inhibiting the release of Ca^2+^ from the SRs may be one of the mechanisms by which dantrolene suppresses the neuroinflammation.

Glutamate is the primary excitatory neurotransmitter in CNS, and one of the glutamate receptors is NMDAR, which plays an important role in excitotoxic injury. Under physiological conditions, once NMDAR are activated, Na^+^ and Ca^2+^ flow will be permitted, which is essential for normal synaptic transmission as well as for a wide range of Ca^2+^- dependent signaling pathways. However, significantly increased glutamate concentration, such as in stroke, leads to loss of ion homeostasis and necrotic cell death by triggering massive NMDAR stimulation.^90^ The elevation of intracellular Ca^2+^ concentration triggers the release of Ca^2+^ from ER through RyRs on ER.^91^ Elevated intracellular Ca^2+^ levels lead to mitochondrial Ca^2+^ overload, which initiates a neurotoxic cascade through multiple mechanisms, including mitochondrial membranes rupture, ROS generation and release of cytochrome c and other proapoptotic factors.^92^ Ultimately these mitochondrial-related events result in necrotic or apoptotic-like cell death.^93^ Furthermore, emerging studies show that abnormal Ca^2+^ release from the ER/SR through RyRs Ca^2+^ channels play an important role in neuroinflammation.^94^

Taken together, dantrolene, a RyRs antagonist acts as a neuroprotective agent through multiple mechanisms proposed below.

### Dantrolene protects neurons by decreasing excitotoxicity

In in vitro experiment on mouse cerebral cortex neurons, dantrolene reduced glutamate-induced increases in intracellular Ca^2+^ by 70% under physiological conditions, protecting neurons from glutamate-induced neurotoxicity.^95^ In another animal experiment, dantrolene significantly reduced the ischemia-induced increase in glutamate concentration and prevented neuronal loss in the CA1 region of the rat hippocampus.^96^ In several other in vitro experiments, dantrolene has been shown to inhibit activation of the NMDA receptors in multiple different cell lines.^97–99^

### Dantrolene protects neurons by decreasing apoptosis

Several animal models have showed neuroprotective effect through apoptosis mechanism. In an in vivo experiment using repeated electroconvulsions as a rat status epilepticus model, the TUNEL method was used to detect cell apoptosis. More TUNEL (+) neurons were observed in the hippocampus of the dantrolene-treated group compared with the control group.^100^ In another rat spinal cord injury model, the author combined riluzole and dantrolene for treatment; they found that rats treated with a combination of riluzole and dantrolene showed a greater number of NeuN-positive neurons, suggesting that dantrolene prevents neuronal apoptosis.^101^

An in vitro experiment using neuron-like PC12 cells demonstrated that dantrolene, as well as 2-aminoethoxydiphenyl borate (2-APB), another RyRs and InsP_3_ receptor (InsP_3_R) inhibitor, protect PC12 cells from H_2_O_2_-induced apoptosis and activate autophagic pathways.^102^

### Dantrolene protects neurons by decreasing neuroinflammation

An increasing number of studies, including different animal models of NDs show that Pro-inflammatory markers are significantly reduced after treatment with dantrolene as we mentioned before. The proposed mechanisms by which dantrolene decreases neuroinflammation include the following approaches.

#### Dantrolene reduces neuroinflammation by inhibiting microglia activation.

Neuroinflammation is characterized by the production of pro-inflammatory cytokines, including IL-1β, IL-6, IL-18 and tumor necrosis factor (TNF), in which microglia play an important role. Once the microglia are activated by pathological factors such as apoptotic neurons or infectious pathogens, they produce increased amounts of pro-inflammatory cytokines.^103^ Furthermore, the insufficient phagocytic capacity of activated microglia leads to the inefficient clearance of protein aggregates such as Aβ in AD.^22^ Activated microglia can also directly eliminate synaptic structures.^103^ The emerging role of microglial activation in the pathogenesis of NDs makes microglia a therapeutic target.

Ca^2+^ is required for LPS-mediated microglia activation in vitro, and the application of Ca^2+^ chelators is sufficient to prevent activation and production of proinflammatory cytokines.^104^ A study has shown that nitrogen-doped graphene quantum dots (N-GQDs) can induce intracellular calcium overload by activating calcium channels of L VGCCs and RyRs, thereby activating mouse hippocampal microglia.^105^ These evidences revealed that RyRs and l-VDCCs participated in mediating Ca^2+^-related microglia activation. Microglia express mRNA for RyR1 and RyR2 isoforms, and application of RyRs antagonists prevents microglia-mediated LPS-induced neurotoxicity.^106^ Another study demonstrated that dantrolene significantly reduced the number of activated microglia in the hippocampus and reduced the expression of various pro-inflammatory cytokines.^73^ Taken together, dantrolene decrease microglia activation by reducing intracellular calcium overload through RyRs channels.

#### Dantrolene reduces neuroinflammation by inhibiting pro-inflammatory cytokines.

As we summarized previously, most NDs are characterized by a pathological inflammatory response. The inflammatory response is initially protective, but subsequently, inflammatory cytokines such as TNFα, IL-6, interleukin 1α(IL-1α), and GM-CSF^22^ lead to cell death and tissue damage.^34^ Intracellular Ca^2+^ signaling and the elevation of intracellular Ca^2+^ are critical for the release of pro-inflammatory cytokines.^71^ Furthermore, calcium channels contribute to the release of pro-inflammatory cytokines in NDs. A recent study showed that knockdown of transient receptor potential vanilloid 4(TRPV4), a nonselective Ca^2+^ channel, can reduce the concentration of pro-inflammatory cytokines including IL-18, COX-2, and 5-LOX in PD mice.^107^ The calcium channel blocker carvedilol has been demonstrated to decrease the release of pro-inflammatory cytokine TNF-α IL-2 and IL-6.^108^ Many research groups also reported that VGCCs and RyRs play an important role in neuroinflammation.^68,109^

Dantrolene, a calcium channel blocker with the ability to ameliorate Ca2 + dysregulation by inhibiting RyRs, has been discovered to suppress plasma and tissue concentration of IL-6, IL-8, IL-1β, TNF-α, and IFN-γ36 in vivo and in vitro.^110–113^ Taken together, dantrolene inhibited ER-mediated Ca^2+^ release and reduced pro-inflammatory cytokine release.

#### Dantrolene reduces neuroinflammation by ameliorating oxidative stress.

Oxidative stress (OS) and neuroinflammation are two different but fundamental pathological factors that play important roles in the onset and progression of NDs.^114^ Inflammatory cells such as microglia and astrocytes secrete reactive substances that promote OS.^114^ Some ROS and RNS (reactive nitrogen species) can reversely stimulate intracellular signaling cascades, resulting in an increased release of pro-inflammatory cytokines. Therefore, neuroinflammation and OS can promote each other, especially in the NDs state.

Given that oxidative stress plays a crucial role in NDs by causing cell death and promoting neuroinflammation, antioxidant drugs have been a potential treatment for NDs. Dantrolene has been reported to protect cells from oxidative stress by increasing the concentration of GSH and GSH/GSSG.^115,116^ Furthermore, calcium influx through RyRs on ER is associated with ROS generation.^117^

#### Dantrolene reduces neuroinflammation by ameliorating pyroptosis.

Pyroptosis is a type of programmed cell death during which the inflammatory cytokines are released. Pyroptosis is mediated by GSDMD-N (N-terminal fragment of gasdermin D) which binds to membranes to form membrane pores and promotes the release of inflammatory cytokines, especially IL-1β. NLRP3 inflammasome activation plays a leading role in the initiation of this process. Pyroptosis is closely related to neuroinflammatory diseases, including subarachnoid hemorrhage, spinal cord injury^118^ and PD.^119^ Recent studies have shown that ER stress and excessive Ca^2+^ release are associated with NLRP3 inflammasome activation. A recent study shows that restriction of Ca^2+^ release in the ER decreases the NLRP3 inflammasome activation.^118^ Dantrolene, as a RyRs antagonist, can also inhibit Ca^2+^ release in the ER and therefore, potentially ameliorate pyroptosis by inhibiting NLRP3 inflammasome activation.

## Future trends in NDs and potential application of dantrolene

In summary, the pathogenesis of NDs primarily involves neuroinflammation and apoptosis mediated by mitochondrial dysfunction. To date there are no disease-modified drugs. On the one hand, dantrolene shows anti-inflammatory effect in a variety of NDs; on the other hand, there are disease-modified drugs for the treatment of AD. All these evidences suggest that dantrolene, as a potential anti-inflammation drug, can be considered as a potential therapeutic drug for the treatment of different NDs.

## Figures and Tables

**Table 1:** Prevalence and major symptoms of common NDs.

Disease	Prevalence	Major Symptoms
Alzheimer’s disease (AD)	6.7 million age 65 and older in the US in 2023^2^	Impairment of learning and memory, speech difficulties
Parkinson’s disease (PD)	2–3 *%* of the global population aged > 65 years in 2017^3^	Muscle tremor, rigidity, bradykinesia/akinesia, and postural instability
Huntington’s disease (HD)	10.6–13.7 per 100,000 in Western countries. 1–7 per million in Japan, Taiwan, and Hong Kong in 2017^4^	Chorea, dystonia, loss of coordination, cognitive decline, behavioral difficulties
Amyotrophic lateral sclerosis (ALS)	Prevalence rates (per 100,000 persons) and incidence rates (per 100,000 person-years) are 6.22 and 2.31 for Europe, 5.20 and 2.35 for North America, 3.41 and 1.25 for Latin America, 3.01 and 0.93 for Asian countries excluding Japan, and 7.96 and 1.76 for Japan, respectively.^5^	Progressive motor defects, with muscle weakness, atrophy and spasms
Multiple sclerosis (MS)	2.8 million people worldwide in 2021^6^	Blurred vision, weak limbs, tingling sensations, unsteadiness, and fatigue

## Data Availability

All study data are included in the article.
